# Spontaneous Bilateral Subdural Hematomas in a Patient With Cryptogenic Liver Cirrhosis

**DOI:** 10.7759/cureus.16100

**Published:** 2021-07-01

**Authors:** Soban Ahmad, Hassam Ali, Sundus Ikram, Shiza Sarfraz, Amman Yousaf

**Affiliations:** 1 Internal Medicine, East Carolina University, Greenville, USA; 2 Internal Medicine, SEGi University, Petaling Jaya, MYS; 3 Anesthesiology, Bahawal Victoria Hospital, Quaid-E-Azam Medical University, Bahawalpur, PAK; 4 Internal Medicine, McLaren Health Care, Flint, USA

**Keywords:** non-traumatic, subdural hematoma, cryptogenic, liver cirrhosis, encephalopathy

## Abstract

Spontaneous subdural hematoma (SDH) in cirrhotic patients is a rarely described condition in the literature and carries a high mortality rate. Several factors can potentially contribute to SDH development in cirrhosis, including coagulation cascade defects, thrombocytopenia, arteriovenous malformations, and cerebral atrophy. Clinicians should always keep spontaneous development of SDH in the differential diagnosis of acute encephalopathy in patients with end-stage liver disease, and prompt head imaging should be considered. We report a unique case of a 64-year-old patient with cryptogenic liver cirrhosis who was found to have spontaneous, bilateral SDHs while undergoing workup for acute encephalopathy.

## Introduction

Subdural hematoma (SDH) is a neurosurgical emergency that is frequently seen in patients with head trauma and carries a high mortality rate of 60%-80% [1]. SDH usually results from injury of the cerebral bridging veins secondary to head trauma resulting in blood collection between the dura mater and the brain parenchyma. The spontaneous or non-traumatic occurrence of acute SDH is an infrequent clinical entity with a reported incidence of 2%-6.7% in the literature [2]. Patients with cirrhosis frequently have low platelet counts and coagulation factor deficiencies, putting them at high risk for spontaneous bleeding [3]. One study was conducted by Lin et al. in Taiwan to investigate the risk for SDHs development in cirrhotic patients [4]. We present a unique case of spontaneous, bilateral, subacute SDHs in a critically ill patient with cryptogenic cirrhosis.

## Case presentation

A 64-year-old Caucasian male with a pertinent medical history of cryptogenic liver cirrhosis, esophageal varices, urinary bladder transitional cell carcinoma status post-cystectomy, and urostomy was transferred to our hospital with hemorrhagic shock due to severe bleeding from the urostomy site. His initial vital signs were as follows: temperature, 36.7°C; blood pressure, 72/50 mmHg; heart rate, 76/min; respiratory rate, 18 breaths/min; and SaO_2_ 98% on four-liter oxygen via nasal cannula. The patient had an unremarkable neurologic examination on presentation. Multiple ecchymoses and petechiae were noticed over both lower extremities. An abdominal exam revealed minimal ascites, and bright red blood was present in the urostomy bag.

**Figure 1:**
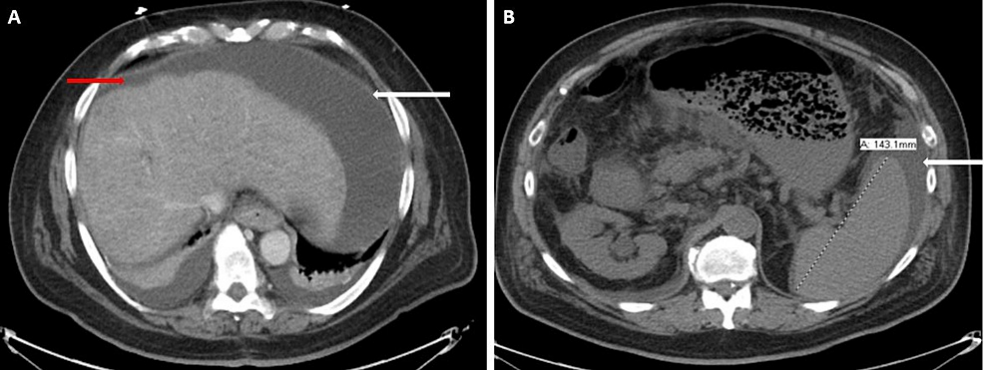
CT abdomen (selected upper abdominal axial sections) Figure 1A demonstrates irregular margins (red arrow) of the liver with nodular appearance and mild-to-moderate amount of free fluid (white arrow). Figure 1B depicts mild splenomegaly (splenic size 14.3 cm) with mild peri-splenic free fluid (white arrow).

His initial laboratory studies revealed hemoglobin 5.2 g/dL, leucocyte count 10.38 x103/µL, platelet 62 x 103/µL, total bilirubin 4.8 mg/dl, aspartate aminotransferase 100 µ/L, alanine aminotransferase 47 µ/L, serum albumin 2.1 g/dl, and serum ammonia level of 128 µmol/L. Coagulopathy was evident with an international normalized ratio of 2.5, prothrombin time of 29.1 (10.2-12.9), partial thromboplastin time of 59.5 (25.1-36.5), and a fibrinogen level of 125 mg/dL. Baseline thrombo-elastography (TEG) test was performed that revealed slightly decreased reaction (R) time 4.1 (5-10 minutes), maximum amplitude (MA) 49.1 (50-70 mm), and lysis at 30 minutes (LY30) of zero percent (0%-30%). Factor VIII percentage was 279% (50%-150%). Disseminated intravascular coagulation was ruled out based on the TEG study findings and factor VIII percentage. The rest of the labs including basic metabolic panel, thyroid function test, lactic acid, troponin, and brain natriuretic peptide (BNP) levels were unremarkable. His workup for cirrhosis including hepatitis B, hepatitis C, autoimmune hepatitis, alpha-1 antitrypsin deficiency, and hemochromatosis was unremarkable. Computed tomography (CT) of the abdomen and pelvis revealed liver cirrhosis, moderate splenomegaly, and mild-to-moderate abdominal ascites (Figures 1A, 1B).

**Figure 2:**
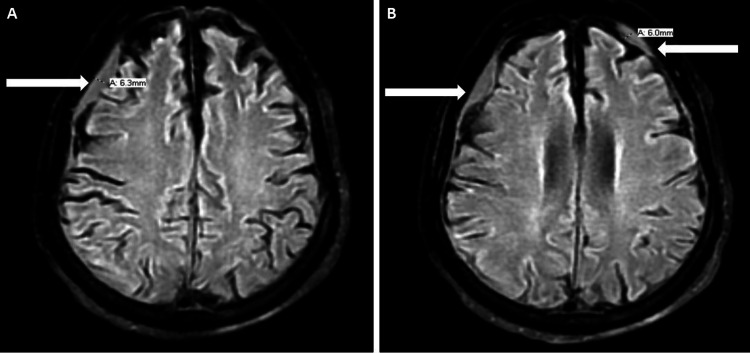
MRI brain T2-weighted axial flair (fluid-attenuated inversion recovery) sequence Figure 2A demonstrates a 6.3-mm rim of subacute subdural hematoma abutting the right frontoparietal lobe (white arrow). Figure 1B shows subtle rims of small, subacute subdural hematomas abutting the bilateral frontal lobes (white arrows).

The patient was aggressively resuscitated with intravenous crystalloids and blood transfusions. Coagulopathy due to liver cirrhosis was corrected with fresh frozen plasma transfusion and vitamin K. Urgent urologic consultation was ordered, and hemostasis was achieved with a single figure of eight-suture placement. Within a few hours, the patient developed a deteriorating mental status requiring intubation for airway protection. The patient underwent a CT scan of the head, which was unremarkable, and he was treated empirically for hepatic encephalopathy with lactulose solution, and serum ammonia level was trended down. Spontaneous bacterial peritonitis was ruled out with paracentesis (ascitic fluid neutrophil count of 91/µL). Electroencephalography showed a diffuse slowing of cortical waves suggestive of severe encephalopathy without any epileptiform discharges. The patient’s neurologic status failed to improve despite getting treatment for hepatic encephalopathy. An MRI (magnetic resonance imaging) of the brain was obtained on post-admit day seven that showed new subacute bilateral SDHs abutting the frontal lobes (Figures 2A, 2B).

Due to multiple comorbidities and an overall dismal prognosis, the patient was not deemed fit for any surgical interventions. He was managed conservatively with the replacement of coagulation factors and platelets. His clinical course remained unchanged without any signs of neurologic recovery. He was placed on comfort measures only by the family, and he died shortly after.

## Discussion

Spontaneous SDH secondary to liver cirrhosis is a rare clinical entity and has not been well reported. Our literature search revealed only one retrospective study conducted by Lin et al. that suggested a possible causal relationship between liver cirrhosis and SDH [4]. This study reported 2.76 folds increased incidence of both traumatic and non-traumatic SDH in cirrhotic patients than the control group with an adjusted hazard ratio of 3.09 for non-traumatic SDH [4]. Most patients with liver cirrhosis have coagulation cascade defects likely contributing toward SDH development. Several factors in patients with liver cirrhosis can contribute toward an increased incidence of SDH (Table 1).

**Table 1:** Contributing factors for subdural hematoma development in patients with liver cirrhosis

Contributing factors	Possible underlying mechanisms
Coagulation factor deficiency	Inadequate production of factors I, II, V, VII, IX, X, and XI [5]. Acquired vitamin K deficiency leading to impaired γ-carboxylation of factors II, VII, IX, and X in the hepatocytes [6].
Thrombocytopenia	Reduced thrombopoietin synthesis and splenic sequestration leading to thrombocytopenia [7,8].
Arteriovenous malformations	Associated with hepatopathy, venous hypertension, increased angiogenesis.
Cerebral atrophy	Concurrent alcohol use, aging, chronic exposure to neurotoxins from liver impairment [8].

The fibrinolytic system is also altered in cirrhotic patients resulting in increased fibrinolysis and premature dissolution of blood clots contributing to coagulopathy [5,9,10]. Although our patient did not use antithrombotic medications before admission, they are the known independent risk factors for spontaneous SDH development. A case-control study reported similar observations in patients using aspirin, warfarin, clopidogrel, and direct oral anticoagulants [11]. In the setting of hemostatic dysfunction secondary to liver cirrhosis, increased fibrinolysis potentially augments the risk of SDH formation.

Liver cirrhosis has been linked to increased incidence of falls, especially in patients with overt hepatic encephalopathy [12,13]. Diffuse cerebral atrophy is also commonly seen in cirrhotic patients, especially those with alcohol use, resulting in the higher vulnerability of bridging vessels to rupture, leading to SDH formation. Increased de-novo arteriovenous malformations (AVMs) in cirrhotic patients might be related to venous hypertension and pro-angiogenic changes resulting from hepatic dysfunction [14]. Rupture of these AVMs at the cortical surface may present with isolated acute SDH [6,15]. Differentiating disseminated intravascular coagulation (DIC) from coagulopathy secondary to liver cirrhosis could be challenging. In our case, initial laboratory abnormalities suggested a possibility of DIC that was ruled out in the absence of hemolysis on TEG (thromboelastography) and elevated factor VIII level [16].

Spontaneous, SDH is a grave disease with a mortality rate reaching as high as 80% in patients presenting with coma [17,18]. Another important prognostic factor is the patient's age, with higher mortality seen in the elderly patients [17]. Patients with liver cirrhosis and acute mental status changes are often treated empirically for hepatic encephalopathy, and brain imaging is deferred in the absence of a history of head trauma. Our case highlights the importance of prompt brain imaging in these patients to rule out spontaneous development of SDH. Although immediate surgical decompression with craniotomy is indicated in most patients with acute traumatic SDH, there are no randomized control trials to address the management of spontaneous, non-traumatic SDH in patients with liver cirrhosis. A few reported cases in the literature demonstrate spontaneous resolution of SDH in cirrhotic patients with conservative management [19,20]. Our patient was also managed conservatively with the correction of an underlying coagulopathy, but overall he had a dismal clinical outcome.

## Conclusions

This case reinforces the previously reported hypothesis that patients with liver cirrhosis are at a higher risk for the development of spontaneous SDH. Because of the unfavorable outcome and a high mortality rate, clinicians should always rule out spontaneous SDH as a possible cause of altered mental status in cirrhotic patients (in addition to hepatic encephalopathy), and prompt head imaging should be performed. Furthermore, we suggest that clinical studies should be carried out to develop specific guidelines about the management of spontaneous SDH in patients with liver cirrhosis.
